# Impact of Nirsevimab on Respiratory Syncytial Virus PCR Test Positivity in Young Infants: A Community‐Level Observational Study in Queensland, Australia

**DOI:** 10.1111/jpc.70317

**Published:** 2026-02-12

**Authors:** Asmaa El‐Heneidy, Keith Grimwood, Jennifer M. Robson, Chanpaseuth Vicki Vilaylath, Sarah G. Cherian, Stephen B. Lambert, Robert S. Ware

**Affiliations:** ^1^ Griffith Biostatistics Unit Griffith University Gold Coast Queensland Australia; ^2^ School of Medicine and Dentistry Griffith University Gold Coast Queensland Australia; ^3^ Sullivan Nicolaides Pathology Bowen Hills Queensland Australia; ^4^ UQ Centre for Clinical Research The University of Queensland Brisbane Queensland Australia; ^5^ Wesley Research Institute Brisbane Queensland Australia

**Keywords:** ecological study, monoclonal antibody, nirsevimab, respiratory syncytial virus

## Abstract

**Aim:**

In April 2024 nirsevimab, a long‐acting respiratory syncytial virus (RSV)‐specific monoclonal antibody, was made available free of charge to all newborn infants in Queensland. We aimed to explore the community‐level impact of a nirsevimab‐based prevention program on RSV detection percentages among infants aged ≤ 3 months in Queensland, Australia.

**Methods:**

A retrospective analysis of January 2022**–**December 2024 laboratory data from a Queensland network. Weekly percentages of RSV‐positive tests among infants aged ≤ 3 months (eligible group for nirsevimab) were compared with children aged 24**–**35 months (ineligible group) after nirsevimab introduction in mid‐April 2024, and with comparable age data from 2022 to 2023.

**Results:**

Overall, 20 956 RSV polymerase chain reaction tests for both age groups were performed during the study period (2022: 5593; 2023: 4910; 2024: 10 453). Following nirsevimab introduction, the percentage of RSV positive tests declined from 16.0% (370/2316) to 5.8% (51/876) among eligible infants (aged ≤ 3 months), compared to 18.6% (1879/10 098) to 14.6% (1117/7666) among ineligible children (aged 24–35 months). Difference‐in‐differences analysis showed an absolute risk difference of −6.9 percentage points (95% confidence interval: −13.2 to −4.1, *p* = 0.03).

**Conclusions:**

We present the first real‐world community‐based evidence on the impact of nirsevimab upon RSV detection percentages in infants aged ≤ 3 months in Queensland, Australia. The nirsevimab‐based prevention program may be contributing to a substantial decline in RSV detection percentages among young infants in the community.

## Key Points

- What is already known on this topic:
  ○ Respiratory syncytial virus (RSV) is a leading cause of hospitalisation and severe respiratory illness in young infants worldwide.
  ○ Nirsevimab reduces medically‐attended RSV lower respiratory infections and hospitalisations in infants in phase III clinical trials.
  ○ Previous real‐world studies, including the only Australian‐based report to date, have focused primarily on RSV‐associated hospitalisations.
- What this paper adds:
  ○ This community‐based ecological study presents the first real‐world evidence on the impact of nirsevimab upon RSV disease in infants aged ≤ 3 months in Queensland, Australia.
  ○ The introduction of nirsevimab in mid‐April 2024 in Queensland coincided with a significant decline in RSV detection percentages in respiratory samples from primary healthcare among nirsevimab‐eligible infants (aged ≤ 3 months), compared with nirsevimab‐ineligible children (aged 24–35 months).

## 1 Introduction

Respiratory syncytial virus (RSV) is an important cause of acute respiratory infections in young children [1, 2]. An Australian birth cohort study found 30%, 60%, and 95% of participants had at least one RSV infection in the first 1, 2, and 3 years of life, respectively [3]. For those aged < 2 years in this birth cohort, 34% of RSV infections resulted in community‐based medical care and 2% in hospitalisation. Indeed, RSV infections are the leading cause of hospitalisation in the first‐year of life with 1.5% of Australian infants admitted annually [4]. Those aged < 3 months are at highest risk of hospitalisation, where at least 65% are born full‐term and otherwise healthy [5].

Nirsevimab (Beyfortus; AstraZeneca and Sanofi) is a long‐acting monoclonal antibody that neutralises RSV and was developed to protect infants from severe RSV infection. In prelicensure trials involving infants aged < 12 months, a single injection of nirsevimab gave a protective efficacy of 74.5% (95% confidence interval [CI], 49.6–87.1) against medically‐attended RSV‐associated lower respiratory infections (LRIs) and 82.7% (95% CI: 67.8–91.5) against hospitalisation for RSV‐associated LRIs for the next 150 and 180 days, respectively [6, 7].

In Australia, nirsevimab was registered by the Therapeutic Goods Administration in November 2023 [8]. The Queensland Government funded and implemented a statewide program from 15 April 2024, making it available year‐round and free of charge to all infants immediately post‐birth, with an option to receive nirsevimab up until 8 months of age in primary care settings if not received in hospital. Children aged < 2 years born < 32 weeks gestation or with complex medical conditions are eligible for a catch‐up dose [9].

Whilst severe outcomes are a primary target for prevention, assessing the impact of nirsevimab against RSV infection in community‐based primary healthcare settings is also important. This is where most mild‐to‐moderate RSV infections are diagnosed and managed. Unlike controlled clinical trials that measure direct nirsevimab efficacy in study participants, ecological studies capture real‐world effects of nirsevimab prevention programs, which are most relevant for public health policy and program evaluation.

Sullivan Nicolaides Pathology (SNP) is a large network of private referral diagnostic laboratories servicing primary healthcare, private hospitals, and residential care facilities throughout Queensland and Northern New South Wales. SNP undertakes approximately 40% of community laboratory testing in Queensland [10]. The primary aim of this study was to assess the impact of nirsevimab among infants ≤ 3 months of age (highest‐risk age group) [5] at the community‐level by measuring changes in RSV detection and detection percentage compared with nirsevimab‐ineligible children aged 24**–**35 months before and after the program introduction.

## 2 Methods

### 2.1 Setting

Queensland covers an area of 1.85 million km^2^ and is located in the northeast of Australia. It has a population of 5.6 million, with 70% residing within 200 km of the capital city, Brisbane, in the south‐east corner of the state [11]. Queensland has three regions that can influence respiratory virus seasonality: North Queensland is tropical, with warm, wet summers and a pronounced wet season (November to April), but dry, warm winters [12]. Central Queensland is subtropical, with warm, humid summers and mild, dry winters. Rainfall varies from year to year and from season to season but is most intensive from December to February [13]. South Queensland ranges from subtropical at the coast to temperate in the interior, with hot humid summers, thunderstorms, and mild winters [14]. These varied climate zones create a complex epidemiological setting where seasonality and scale of RSV epidemics vary significantly across these regions [15, 16].

### 2.2 Study Design

A retrospective analysis of data from routine diagnostic testing was performed. Data for all RSV polymerase chain reaction (PCR) assay tests on respiratory specimens by SNP laboratories in Queensland from 01 January 2022 to 31 December 2024 were extracted. RSV PCR assays remained unchanged during the study period. Non‐Queensland residents' data were excluded.

### 2.3 Statistical Analysis

Descriptive statistics were used to summarise RSV epidemiology. The year 2024 was divided into pre‐ and post‐nirsevimab periods based upon the government funding for nirsevimab (15 April 2024). A difference‐in‐differences linear probability model was used to compare changes in the percentage of RSV PCR assays that tested positive between the pre‐ and post‐nirsevimab periods for eligible (≤ 3 months) and ineligible (24**–**35 months) age groups. Models were adjusted for month and year of specimen collection to control for seasonality and secular trends. Results are presented as adjusted absolute risk differences (ARD) in percentage points with their corresponding 95% CI.

Cumulative positive detections of RSV PCR tests are presented in the Figure to compare trends from 2022 to 2024 for both key age groups (ages ≤ 3 and 24**–**35 months).

Sensitivity analyses investigated whether the community‐level effects of nirsevimab varied among age subgroups within the eligible group by stratifying according to age at testing for RSV 1‐month, 2 months and 3 months. Analyses were also extended to those aged 4–≤ 6 months. A chi‐square test was conducted to compare the weekly percentage of RSV PCR positive tests in the post‐nirsevimab period in 2024 with a combined pre‐nirsevimab group among age subgroups. The combined pre‐nirsevimab period group included the corresponding calendar periods in 2022 and 2023 as well as the pre‐nirsevimab period in 2024. This approach allowed us to evaluate whether the proportion of detected RSV cases significantly changed following the introduction of nirsevimab, while accounting for seasonal patterns and recent baseline data in 2024. Results are presented as the weekly percentages of all positive RSV PCR tests. Stata v14.2 (StataCorp, College Station, TX, USA) was used to perform all analyses.

Griffith University Human Research Ethics Committee (2024/820) approved the study.

## 3 Results

Overall, 20 956 RSV tests in the study population (3192 aged ≤ 3 months and 17 764 aged 24**–**35 months) were performed from January 2022 to December 2024. The number of RSV tests was highest in 2024 for both age groups (1368/3192 [42.9%] for infants aged ≤ 3 months and 7666/17 764 [43.2%] for children aged 24**–**35 months). Characteristics of tested individuals, the total number of specimens tested, and the number of positive tests from 2022 to 2024 are detailed in Table 1.

**TABLE 1:** Characteristics of infants aged ≤ 3 months and children aged 24–35 months tested for respiratory syncytial virus by year (2022–2024) (*n* = 20 956).

Characteristics	2022	2023	2024	
	01 Jan to 31 Dec	01 Jan to 31 Dec	01 Jan to 14 Apr	15 Apr to 31 Dec
Infants aged ≤ 3 months	(*n* = 994)	(*n* = 830)	(*n* = 492)	(*n* = 876)
	*n* (%)^a^	*n* (%)	*n* (%)	*n* (%)
Number of detections	150 (15.1)	130 (15.7)	90 (18.3)	51 (5.8)
Weekly percentage of positive tests, Mean (SD)	13.2 (16.8)	15.3 (17.7)	13.2 (23.0)	8.7 (20.8)
Sex (male)	538 (54.1)	453 (54.6)	259 (52.6)	463 (52.9)
Region				
North QLD	151 (15.2)	161 (19.4)	86 (17.5)	158 (18.0)
Central QLD	315 (31.7)	264 (31.8)	156 (31.7)	294 (33.6)
South QLD	528 (53.1)	405 (48.8)	250 (50.8)	424 (48.4)

Characteristics	2022	2023	2024	
	01 Jan to 31 Dec	01 Jan to 31 Dec	01 Jan to 14 Apr	15 Apr to 31 Dec
Children aged 24–35 months	(*n* = 4599)	(*n* = 4080)	(*n* = 1419)	(*n* = 7666)
	*n* (%)^a^	*n* (%)	*n* (%)	*n* (%)
Number of detections	872 (19.0)	737 (18.1)	270 (19.0)	1117 (14.6)
Mean weekly percentage of positive tests (SD)	14.0 (15.8)	15.6 (12.2)	17.5 (8.4)	14.1 (6.1)
Sex (male)	2439 (53.0)	2203 (54.0)	752 (53.0)	4013 (52.3)
Region				
North QLD	692 (15.0)	671 (16.4)	241 (17.0)	1365 (17.8)
Central QLD	1453 (31.6)	1361 (33.4)	449 (31.6)	2551 (33.3)
South QLD	2454 (53.3)	2048 (50.2)	729 (51.4)	3750 (48.9)

Abbreviations: *n* (%), number (percentage); QLD, Queensland; SD, standard deviation.

^a^ Percentages may not add to 100 because of rounding.

Most tests were conducted in the Southern (50.3% and 50.6%), followed by the Central (32.2% and 32.7%) and Tropical (17.5% and 16.7%) regions for the ≤ 3‐month and 24**–**35 months age groups, respectively (Table 1).

Between 2022 and 2024 inclusive, 421/3192 (13.2%) and 2996/17764 (16.9%) infants in the ≤ 3 months and 24**–**35 months age groups, respectively, tested positive for RSV. Across the periods 2022, 2023, 2024 pre‐nirsevimab and 2024 post‐nirsevimab, the percentage of positive RSV tests for the ≤ 3 months and 24**–**35 months age groups were 15.1%, 15.7%, 18.3% and 5.8%, and 19.0%, 18.1%, 19.0% and 14.6%, respectively (Table 1). When combining the pre‐nirsevimab periods, the percentage of RSV positive tests among infants aged ≤ 3 months declined from 16.0% (370/2316) to 5.8% (51/876) (10.2 percentage point reduction), compared with a reduction from 18.6% (1879/10 098) to 14.6% (1117/7666) (4.0 percentage point reduction) in children aged 24–35 months. The adjusted ARD of −6.9 percentage points (95% CI: −13.2 to −4.1, *p* = 0.03) indicates infants aged ≤ 3 months experienced an additional 6.9 percentage point reduction in the percentage of tests positive for RSV beyond the background temporal trend (Table 2).

**TABLE 2:** Percentage of respiratory syncytial virus detections pre‐ and post‐nirsevimab introduction (01 January 2022 to 14 April 2024 inclusive versus 15 April to 31 December 2024).

Age group	≤ 3 months (*n* = 3192)	24–35 months (*n* = 17 764)	Difference (pp)
Pre‐nirsevimab	370/2316 (16.0%)	1879/10098 (18.6%)	−2.6
Post‐nirsevimab	51/876 (5.8%)	1117/7666 (14.6%)	−8.8
Absolute change (pp)	−10.2	−4.0	−6.2
Relative reduction	63.8%	21.5%	42.3%
Risk difference^a^ (95% CI)			**−6.9 (−13.2 to −4.1)**

Abbreviations: CI, confidence interval; pp., percentage points.

^a^ Adjusted for calendar month and year of RSV tests. Values in **Bold** indicate statistically significant difference at *p* = 0.03.

**FIGURE 1:**
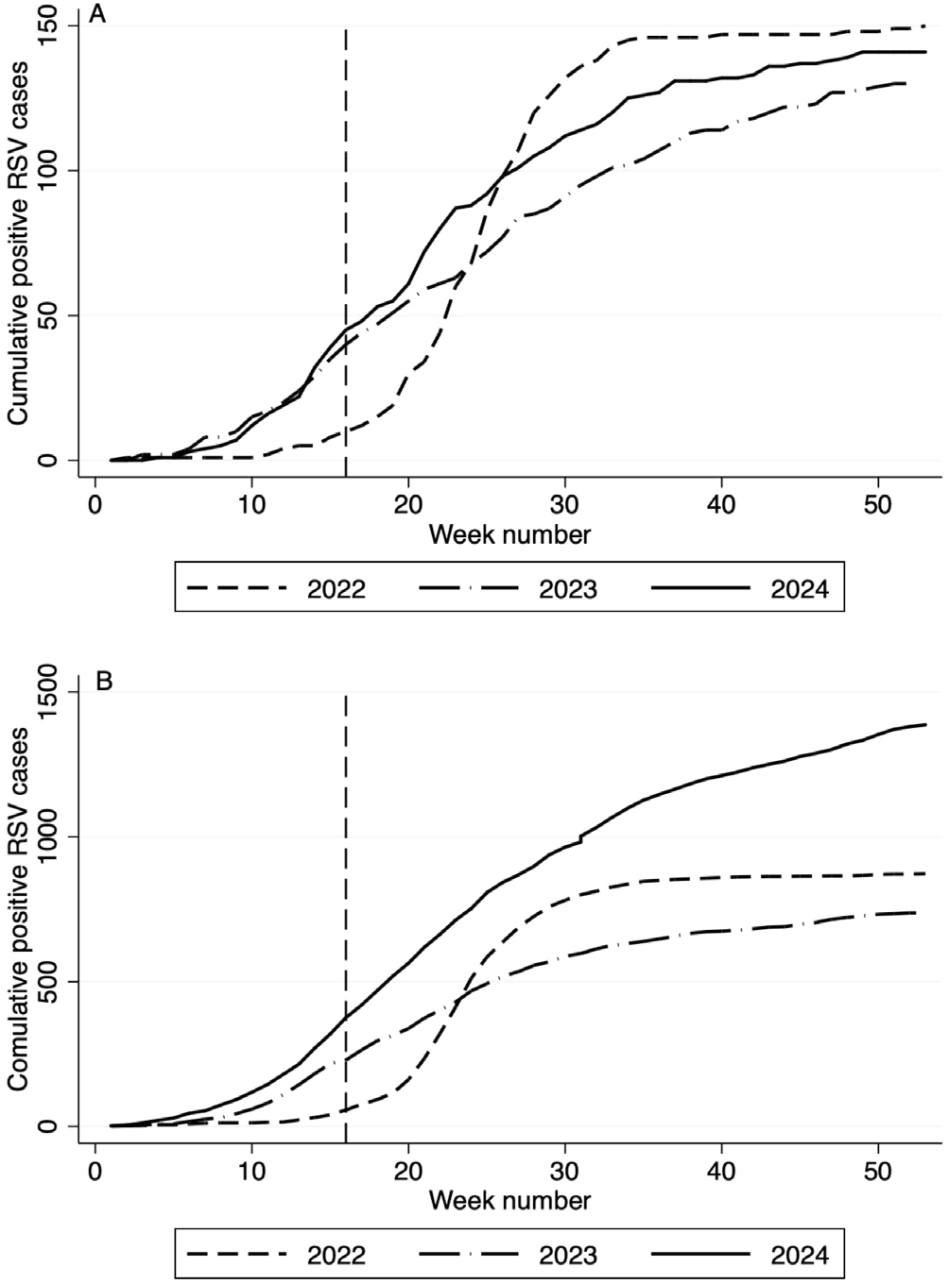
(A) Cumulative positive tests for respiratory syncytial virus in 2022–2024 for infants aged ≤ 3 months. (B) Cumulative positive tests for respiratory syncytial virus in 2022–2024 for children aged 24–35 months. Nirsevimab was introduced on 15 April 2024 (dashed vertical line).

Yearly cumulative RSV positive cases varied across years in both age groups (Figure 1). In 2024, when nirsevimab was introduced, cumulative positives in infants ≤ 3 months of age increased modestly to 141 from 130 in 2023 (8.5% increase). In contrast, cumulative positive cases in children aged 24–35 months increased substantially in 2024 to 1387 from 737 in 2023 (88.2% increase).

When month‐by‐month age at testing was considered, RSV detection percentages during the mid‐April–December periods in 2022 and 2023 were relatively stable with no significant difference between the 2‐years for both age groups (Table 3). The percentage of RSV detections among 1‐month‐old infants was significantly lower in the 2024 post‐nirsevimab period (5.1%) than in the combined 2024 pre‐nirsevimab period and equivalent periods in 2022–2023 (15.4%), representing a 67.0% relative reduction. Infants aged 2 and 3 months showed similar patterns, with post‐nirsevimab detection percentages significantly declining from 15.4% to 6.3% (59.1% relative reduction) and from 12.5% to 6.2% (50.4% relative reduction), respectively. However, a slight decline in RSV detection percentages was also observed among children aged 24**–**35 months in the post‐nirsevimab period from 18.4% to 14.6% (20.7% relative reduction). The percentage positives by Queensland regions are displayed in Figure S1. Repeating the analyses in infants aged 4**–≤** 6 months found a small and non‐significant reduction in RSV detections when compared with children aged 24–35 months (adjusted ARD = −2.9 [95% CI −9.2 to 3.6]) (Tables S1–S3).

**TABLE 3:** Number of tests and tests positive for respiratory syncytial virus in infants aged ≤ 3 months and children aged 24–35 months across two time periods in 2024 and the equivalent periods in 2022–2023, Queensland, Australia.

Year of detection	2022		2023		2024		*p*‐Value^a^
	(Weeks 1–15)	(Weeks 16–53)	(Weeks 1–15)	(Weeks 16–53)	(Weeks 1–15)	(Weeks 16–53)	
Infants aged ≤ 1‐month							
Number of tests	33	319	52	233	79	333	
Number of detections (%)	2 (6.1)	53 (16.6)	7 (13.5)	33 (14.2)	11 (13.9)	17 (5.1)	**< 0.001**

Year of detection	2022		2023		2024		*p*‐Value^a^
	(Weeks 1–22)	(Weeks 23–53)	(Weeks 1–22)	(Weeks 23–53)	(Weeks 1–22)	(Weeks 23–53)	
Infants aged 2 months							
Number of tests	75	209	95	143	140	269	
Number of detections (%)	12 (16.0)	23 (11.0)	21 (22.1)	18 (12.6)	35 (25.0)	17 (6.3)	**0.001**

Year of detection	2022		2023		2024		*p*‐Value^a^
	(Weeks 1–28)	(Weeks 29–53)	(Weeks 1–28)	(Weeks 29–53)	(Weeks 1–28)	(Weeks 29–53)	
Infants aged 3 months							
Number of tests	149	209	165	142	273	274	
Number of detections (%)	41 (27.5)	19 (9.1)	36 (21.8)	15 (10.6)	44 (16.1)	17 (6.2)	**0.01**

Year of detection	2022		2023		2024		*p*‐Value^a^
	(Weeks 1–15)	(Weeks 16–53)	(Weeks 1–15)	(Weeks 16–53)	(Weeks 1–15)	(Weeks 16–53)	
Children aged 24–35 months							
Number of tests	365	4234	696	3384	1419	7666	
Number of detections (%)	31 (8.5)	841 (19.9)	182 (26.1)	555 (16.4)	270 (19.0)	1117 (14.6)	**< 0.001**

^a^ Chi‐squared test was used to compare the percentage of positive tests for RSV in the post‐nirsevimab period of 2024 with a combined pre‐nirsevimab group (2022–2023 matching months and early 2024). Values in **Bold** indicate statistically significant difference at *p* < 0.05.

## 4 Discussion

The introduction of nirsevimab in mid‐April 2024 in Queensland coincided with notable changes in weekly RSV detection percentages among infants aged ≤ 3 months. These findings suggest the nirsevimab‐based RSV prevention program led to a significant decline in RSV detection percentages among infants aged ≤ 3 months tested within community settings at the population level.

Given the limited community contacts and transmission role of infants ≤ 3 months of age, the observed decrease in the percentage of RSV‐positive infants is likely due to the direct protective impact of nirsevimab rather than potential indirect effects. Even though the ecological design means the study cannot isolate these mechanisms or identify individual infants who received nirsevimab [17], these results help determine the real‐world disease burden reduction that is possible when preliminary estimates indicate coverage of 70%–80% in Queensland during the study period (Roughan W and Griffin P, unpublished data, 2025). Further reductions in disease burden are also likely with increased nirsevimab coverage during infancy.

We observed a substantial increase in the total number of PCR tests performed for RSV in 2024 compared to 2022–2023. This may be due to increased testing volumes for *Mycoplasma pneumoniae* and *Bordetella pertussis* , as Queensland experienced a significant surge in pertussis in 2024, with almost a 23‐fold increase in detections to 30‐September compared to the same period in 2023 [18]. This was accompanied by a rise in *M. pneumoniae* cases [19]. Given the increase in testing during 2024, an ineligible age group for nirsevimab (24–35 months) was used to evaluate the nirsevimab impact independently of testing volume.

The strengths of our study are its state‐wide coverage with a strong community focus involving primary healthcare settings and its use of a large prospectively‐collected dataset. Previous studies, including the only Australian‐based report published to date, have focused primarily on RSV‐associated hospitalisations, with limited evaluation of nirsevimab's impact at the community level, which represents a substantial proportion of the RSV burden [20, 21]. Our study evaluated the potential effect of the Queensland nirsevimab‐based RSV prevention program on the percentage of tests positive for RSV month by month in the first 3 months of life, rather than combining age groups. This may allow for more precise clinical guidance when protection is most needed as RSV causes the most severe illness during these time points, with the highest rates of hospitalisation [4, 22], including intensive care unit admission and mechanical ventilation [23, 24].

The study does however have limitations, including the absence of data on receiving nirsevimab at the individual level, which prevents establishing a stronger causal association between nirsevimab program introduction and observed changes in RSV disease metrics. Future RSV reduction in young infants will also be influenced by the introduction of maternal RSV vaccine (Abrysvo, Pfizer) in Queensland in December 2024. Our study did not account for other confounding factors, such as variations in healthcare access and physician testing behaviour, which may have influenced the results, although we did include an older non‐eligible group to control for secular trends. Furthermore, it likely underestimated the impact of the nirsevimab program within the community as it did not capture affected infants managed solely at home by their parents/caregivers or those who were not tested for RSV infection. Despite these limitations, the findings provide valuable insights into disease patterns and the potential impact of a nirsevimab‐based prevention program at reducing RSV detection percentages among infants aged ≤ 3 months managed within the community.

## Author Contributions

Conceptualisation: Asmaa El‐Heneidy, Keith Grimwood, Stephen B. Lambert and Robert S. Ware. Collected and provided the laboratory data: Jennifer M. Robson, Chanpaseuth Vicki Vilaylath and Sarah G. Cherian. Methodology: Asmaa El‐Heneidy and Robert S. Ware. Software: Asmaa El‐Heneidy. Formal analysis: Asmaa El‐Heneidy. Writing – initial draft: Asmaa El‐Heneidy. Writing – review and editing: Keith Grimwood, Jennifer M. Robson, Chanpaseuth Vicki Vilaylath, Sarah G. Cherian, Stephen B. Lambert and Robert S. Ware and all authors approved the final manuscript as submitted and agree to be accountable for all aspects of the work.

## Funding

A.E.‐H., K.G. and R.S.W. have received funding from Sanofi and AstraZeneca through a collaboration grant for unrelated work. K.G. is a recipient of several Australian National Health and Medical Research Council and Medical Research Future Fund grant awards, none of which are related to the subject of this study. He has received lecture fees from Sanofi‐Aventis Australia Pty Ltd.

## Conflicts of Interest

The authors declare no conflicts of interest.

## Supporting information


**Data S1:** jpc70317‐sup‐0001‐Supinfo.docx.

## Data Availability

The data that support the findings of this study are available on request from the corresponding author. The data are not publicly available due to privacy or ethical restrictions.
