# Supplementary material for: Impact of Nirsevimab on Respiratory Syncytial Virus PCR Test Positivity in Young Infants: A Community‐Level Observational Study in Queensland, Australia
Source: J Paediatr Child Health. 2026 Feb 12;62(4):614–20. doi: 10.1111/jpc.70317 (PMC13045763; doi:10.1111/jpc.70317)
Supplement: Supplementary file 1 — Data S1: jpc70317‐sup‐0001‐Supinfo.docx. [file JPC-62-614-s001.docx]

**Supplementary file**

**Impact of nirsevimab on respiratory syncytial virus PCR test positivity in young infants: A community-level observational study in Queensland, Australia**

| Supplementary Table-1. Characteristics of tested children aged 4–<6 months for respiratory syncytial virus by year (2022–2024) (n=5,113). | | | | | |
| --- | --- | --- | --- | --- | --- |
|  | **2022** | **2023** | **2024** | |  |
| Characteristics | 01 Jan to 31 Dec | 01 Jan to 31 Dec | 01 Jan to 14 Apr | 15 Apr to 31 Dec |  |
|  | **(n=1,479)** | **(n=1,307)** | **(n=1,718)** | **(n=609)** |  |
|  | **n (%)^a^** | **n (%)** | **n (%)** | **n (%)** |  |
| Number of detections | 234 (15.8) | 220 (16.8) | 286 (16.6) | 48 (7.9) |  |
| Weekly percentage of positive tests, Mean (SD) | 12.5 (17.3) | 16.3 (17.3) | 15.9 (14.0) | 7.1 (9.2) |  |
| Sex (male) | 796 (53.8) | 706 (54.0) | 938 (54.6) | 348 (57.1) |  |
| Region |  |  |  |  |  |
| North QLD | 241 (16.3) | 234 (17.9) | 322 (18.7) | 149 (24.5) |  |
| Central QLD | 477 (32.3) | 432 (33.1) | 526 (30.6) | 196 (32.2) |  |
| South QLD | 761 (51.5) | 641 (49.0) | 870 (50.6) | 264 (43.3) |  |

**Abbreviation**: n (%), number (percentage); QLD, Queensland; SD, standard deviation. ^a^Percentages may not add to 100 because of rounding.

**Supplementary Table-2. Percentage of detections of respiratory syncytial virus pre- and post-nirsevimab introduction in infants aged 4–<6 months and children aged 24–35 months, respectively.**

| Age group | 4–<6 months  (n=5,113) | 24–35 months (n=17,764) | Difference  (pp) |
| --- | --- | --- | --- |
| Pre-nirsevimab | 740/4,504  (16.4%) | 1,879/10,098 (18.6%) | -2.2 |
| Post-nirsevimab | 48/609  (7.9%) | 1,117/7,666  (14.6%) | -6.7 |
| Absolute change (pp) | -8.5 | -4.0 | -4.5 |
| Relative reduction | 51.8% | 21.5% | 30.3% |
| Risk difference^a^  (95% CI) |  |  | -2.9  (-9.2 to 3.6) |

**Abbreviations:** CI, confidence interval; pp, percentage points; ^a^ adjusted for calendar month and year of RSV tests.

| **Supplementary Table-3. Number of tests and tests positive for respiratory syncytial virus in infants aged 4–<6 months across two time periods in 2024 and the equivalent periods in 2022–2023, Queensland, Australia.** | | | | | | | |
| --- | --- | --- | --- | --- | --- | --- | --- |
| **Year of detection** | **2022** | | **2023** | | **2024** | | **p-value^a^** |
| **Infants aged 4-month** | | | | | | |  |
|  | **(Weeks 1–33)** | **(Weeks 34–53)** | **(Weeks 1–33)** | **(Weeks 34–53)** | **(Weeks 1–33)** | **(Weeks 34–53)** |  |
| **Number of tests** | 245 | 169 | 185 | 147 | 397 | 232 |  |
| **Number of detections (%)** | 54 (22.0) | 10 (5.9) | 42 (22.7) | 16 (10.9) | 68 (16.8) | 17 (7.3) | **0.03** |
| **Infants aged 5-months** | | | | | | | |
|  | **(Weeks 1–37)** | **(Weeks 38–53)** | **(Weeks 1–37)** | **(Weeks 38–53)** | **(Weeks 1–37)** | **(Weeks 38–53)** |  |
| **Number of tests** | 393 | 110 | 328 | 129 | 576 | 219 |  |
| **Number of detections (%)** | 72 (18.3) | 2 (1.8) | 72 (22.0) | 14 (10.9) | 97 (16.8) | 17 (7.8) | **0.03** |
| **Infants aged 6-months** | | | | | | | |
|  | **(Weeks 1–41)** | **(Weeks 42–53)** | **(Weeks 1–41)** | **(Weeks 42–53)** | **(Weeks 1–41)** | **(Weeks 42–53)** |  |
| **Number of tests** | 470 | 92 | 421 | 97 | 745 | 158 |  |
| **Number of detections (%)** | 92 (19.6) | 4 (4.3) | 69 (16.4) | 7 (7.2) | 121 (16.2) | 14 (8.9) | 0.11 |

^a^ Chi-squared test was used to compare the percentage of positive tests for RSV in the post-nirsevimab period of 2024 with a combined pre- nirsevimab group (2022–2023 matching months and early 2024). Values in **Bold** indicate statistically significant difference at p<0.05.


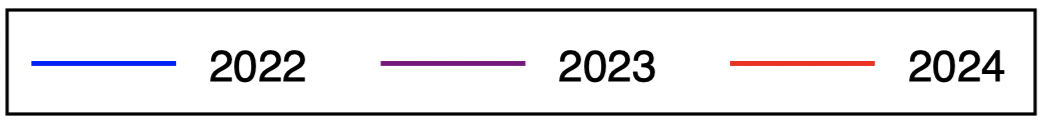


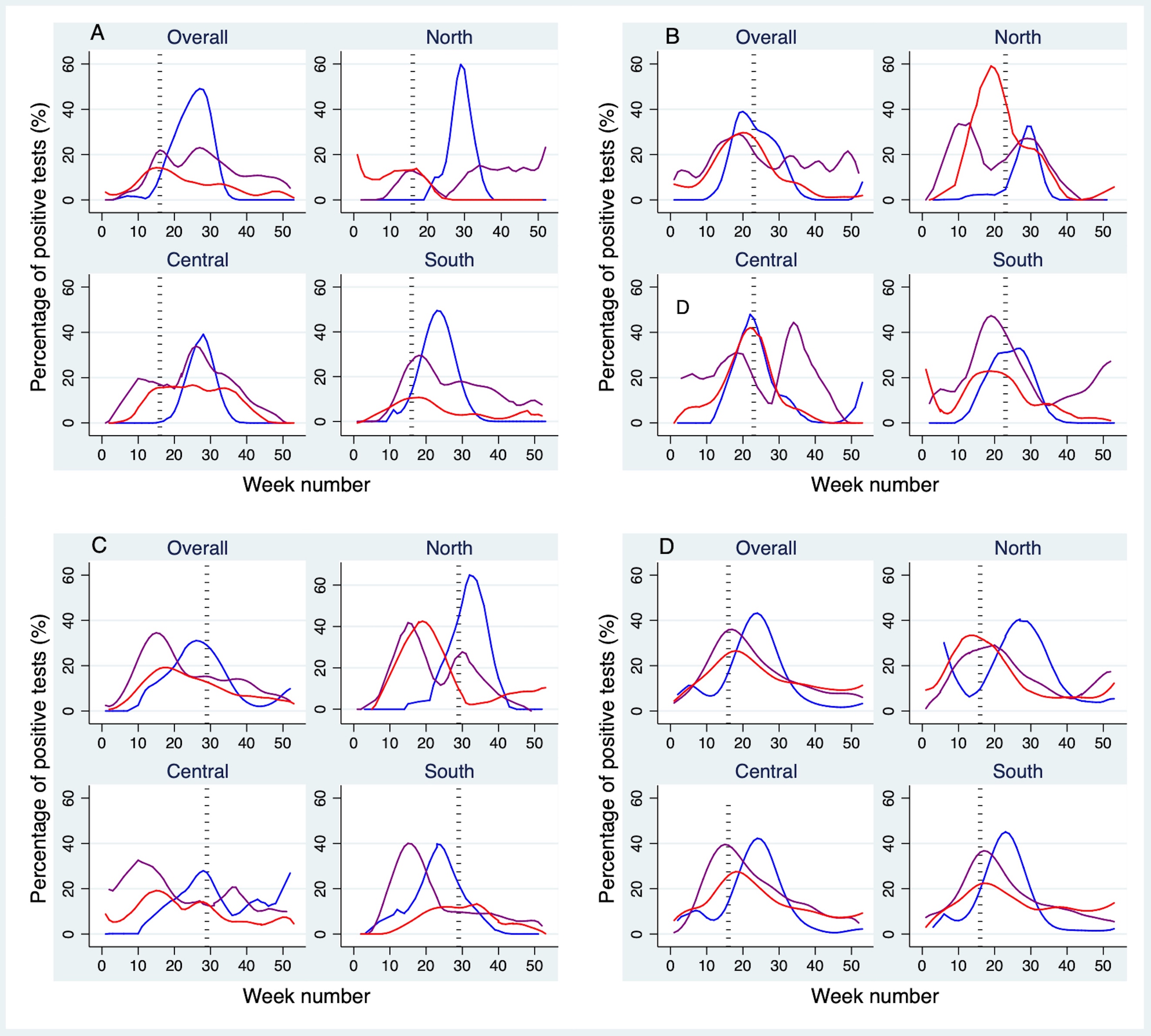


**Supplementary Figure:** Overall and regional Queensland positive respiratory syncytial virus tests in 2022–2024 expressed as weekly percentages for infants and children aged
**A:** ≤1-month. **B:** 2-months. **C:** 3-months. **D:** 24–35 months.

**Note:** The x-axis shows week number of specimen collection. The year 2024 in each panel was divided into two time periods based on the age of infants when tested for respiratory syncytial virus (dashed vertical line).

The vertical line in each panel shows the earliest date of specimen collection when nirsevimab-eligible infants could be tested at that age. Nirsevimab eligibility was determined retrospectively by calculating the date of birth (specimen collection date minus age) and comparing it to the date of nirsevimab introduction (15^th^ of April 2024).

**Figure A** (≤1-month): nirsevimab-eligible infants can be tested immediately after the nirsevimab rollout.

**Figure B** (2-months): nirsevimab-eligible infants born 15^th^ of April 2024 enters their 2nd month of life on 15^th^ of May 2024.

**Figure C** (3-months): nirsevimab-eligible infants born 15^th^ of April 2024 enters their 3rd month of life on 15^th^ of June 2024.
